# Supplementary material for: Combinatorial logic devices based on a multi-path active ring circuit
Source: Sci Rep. 2022 Jun 8;12:9482. doi: 10.1038/s41598-022-13614-2 (PMC9177788; doi:10.1038/s41598-022-13614-2)
Supplement: Supplementary file 1 — Supplementary Information 1. [file 41598_2022_13614_MOESM1_ESM.docx]

**Supplementary Material**

The supplementary material section contains data to support Examples 2 and Example 3.
