# Supplementary material for: Combinatorial logic devices based on a multi-path active ring circuit
Source: Sci Rep. 2022 Jun 8;12:9482. doi: 10.1038/s41598-022-13614-2 (PMC9177788; doi:10.1038/s41598-022-13614-2)

Supplementary Materials

Schematics of all possible paths connecting input 1 and output 1

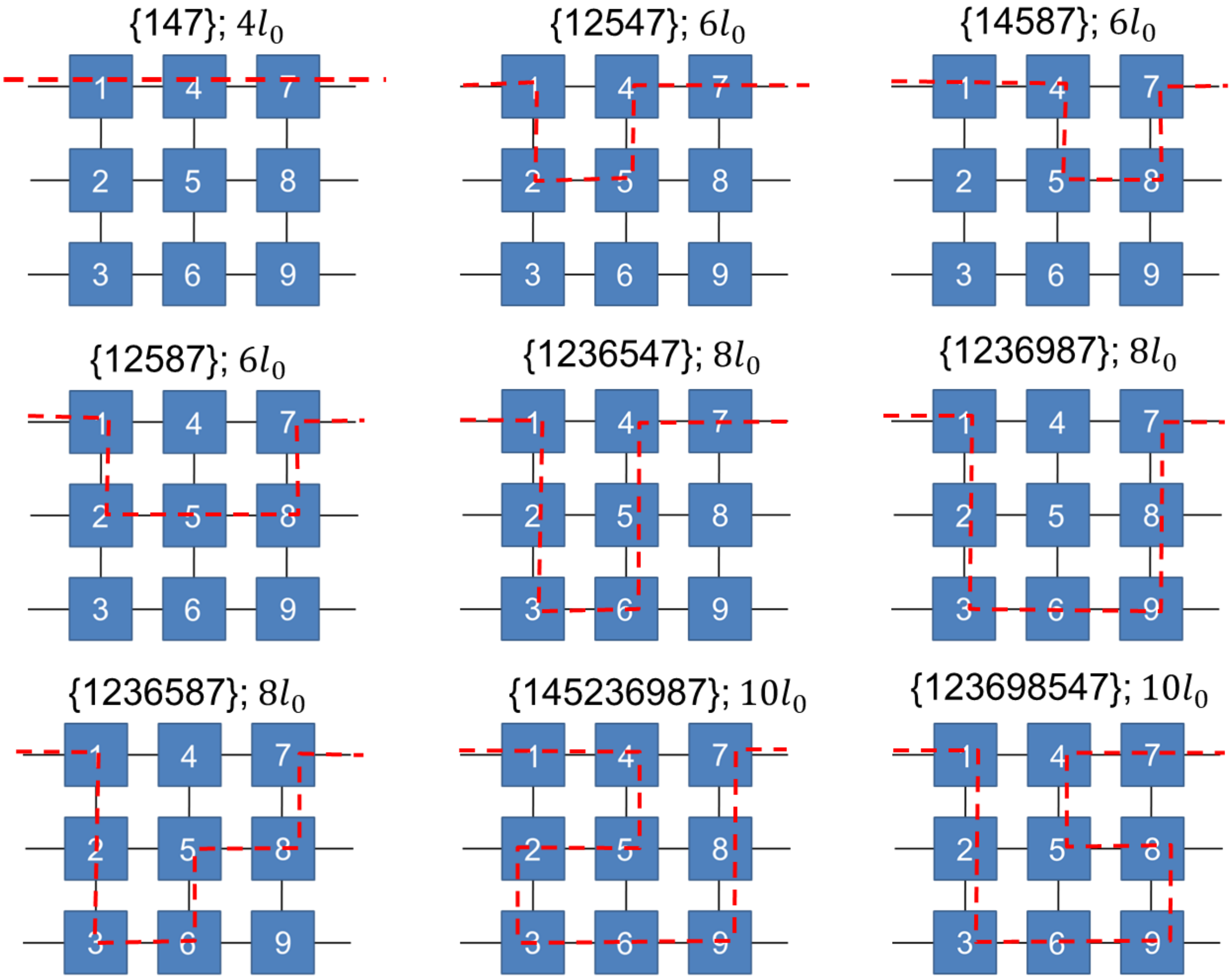

Schematics of all possible paths connecting input 1 and output 2

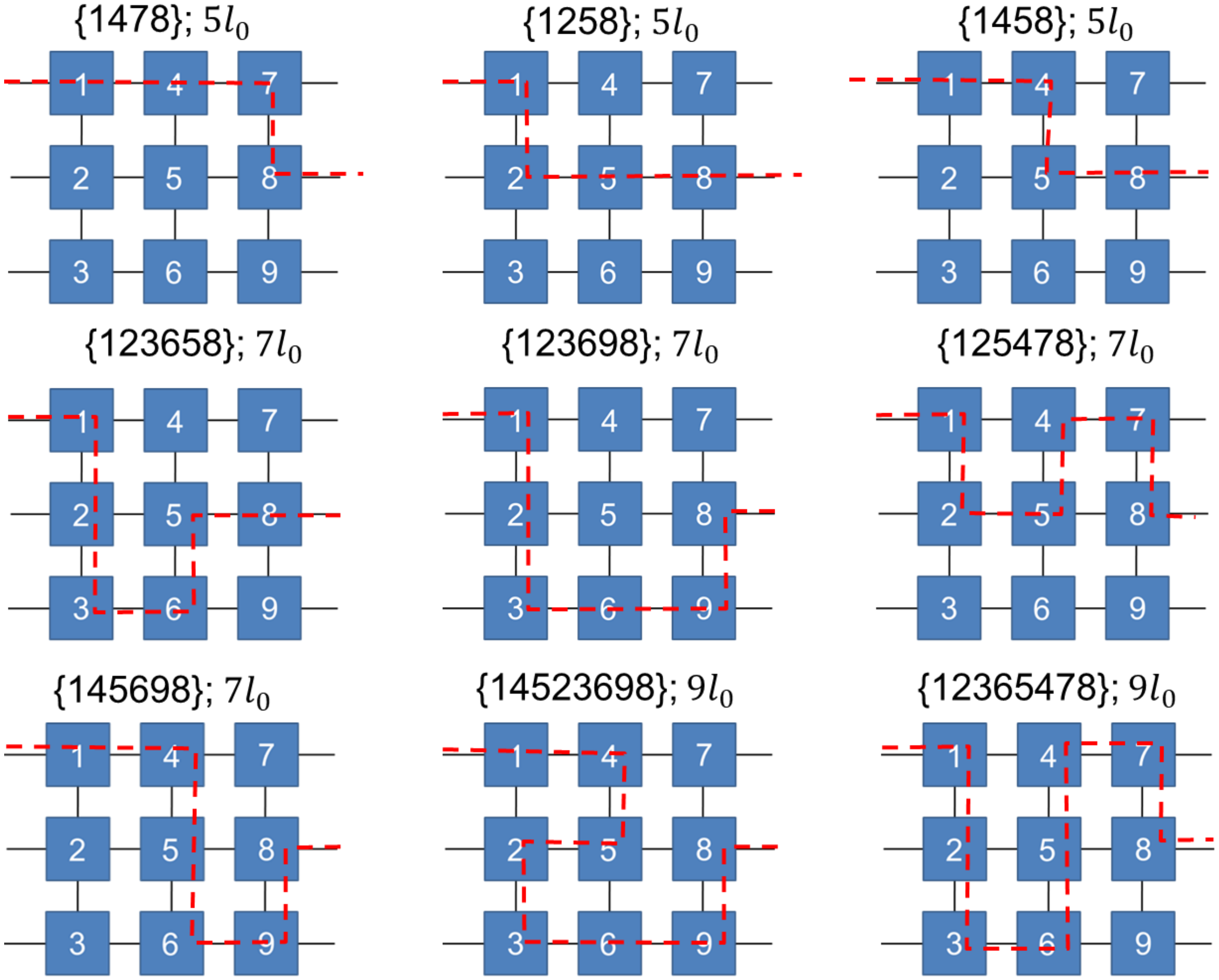

Schematics of all possible paths connecting input 1 and output 3

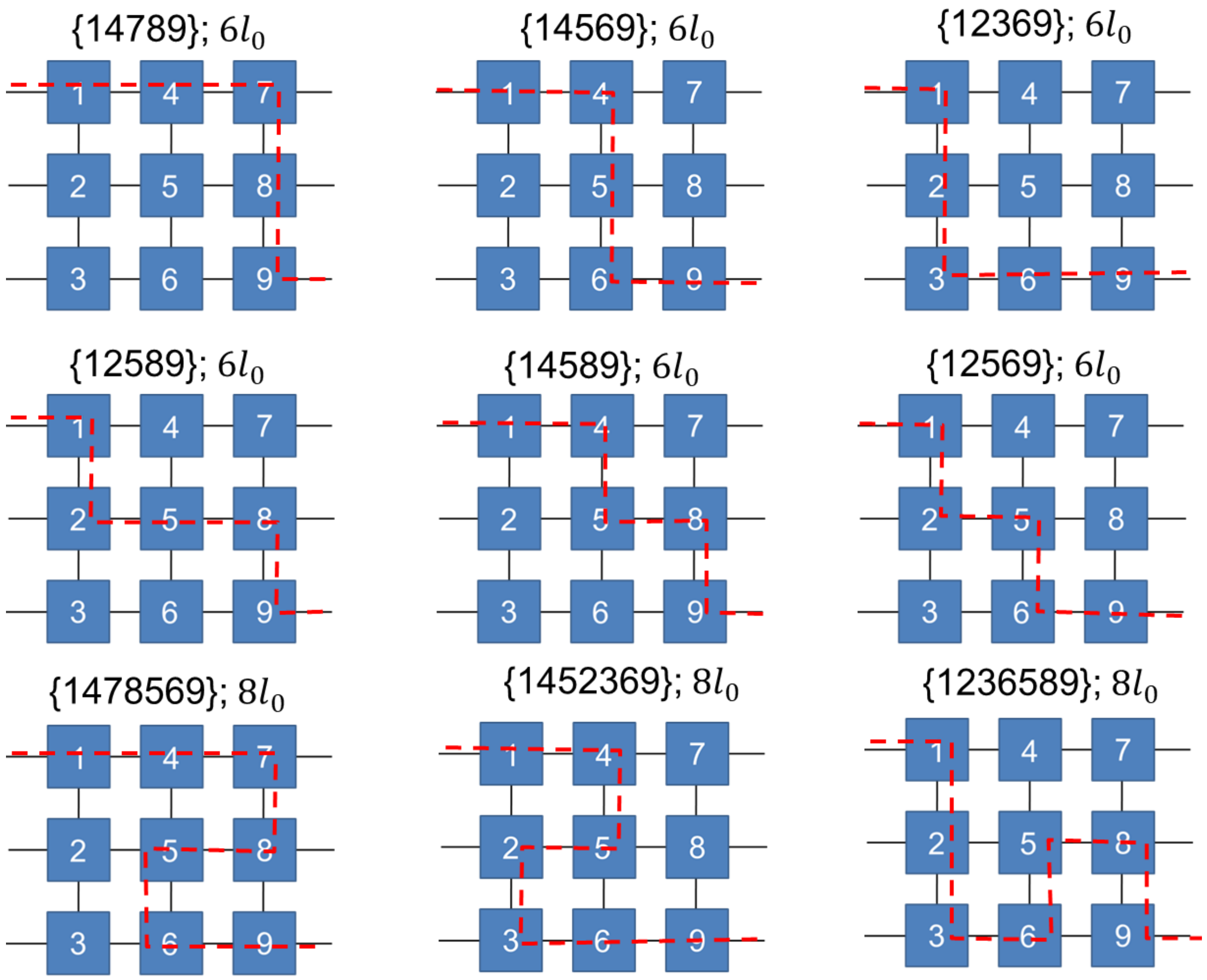

Schematics of all possible paths connecting input 1 and output 3 cont'd

{123654789}; $10l_0$

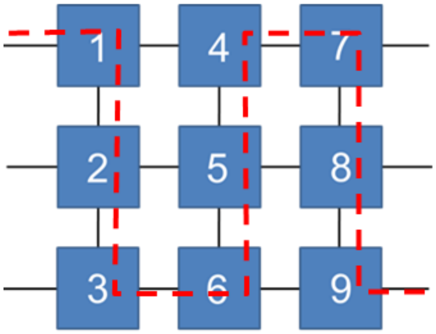

{147852369}; $10l_0$

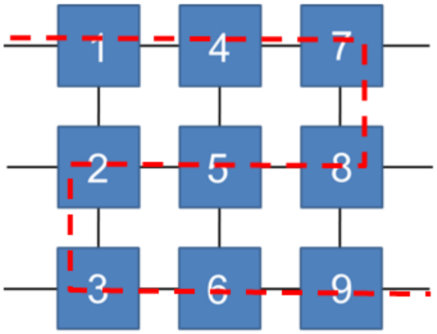

Schematics of all possible paths connecting input 2 and output 1

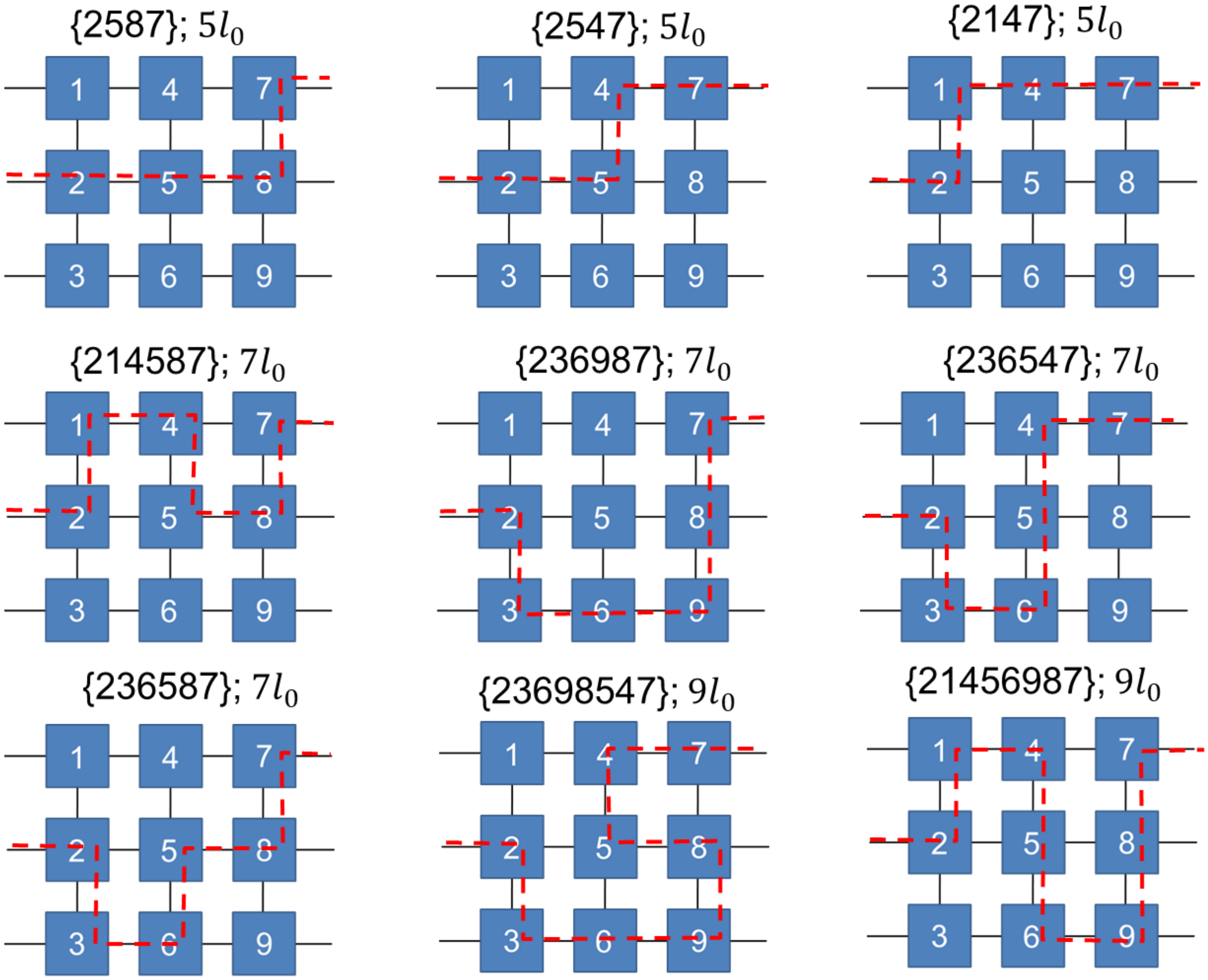

Schematics of all possible paths connecting input 2 and output 2

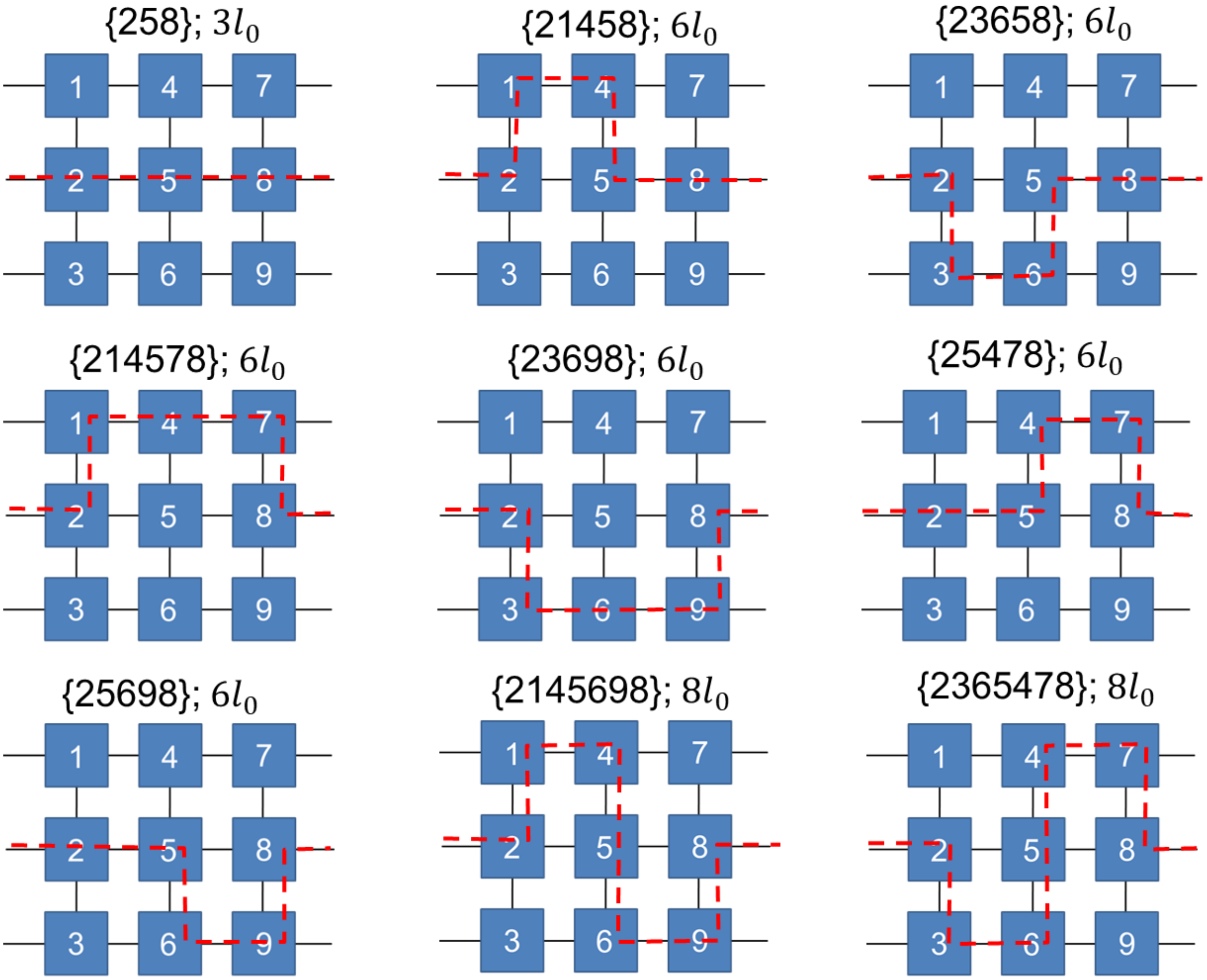

Schematics of all possible paths connecting input 2 and output 3

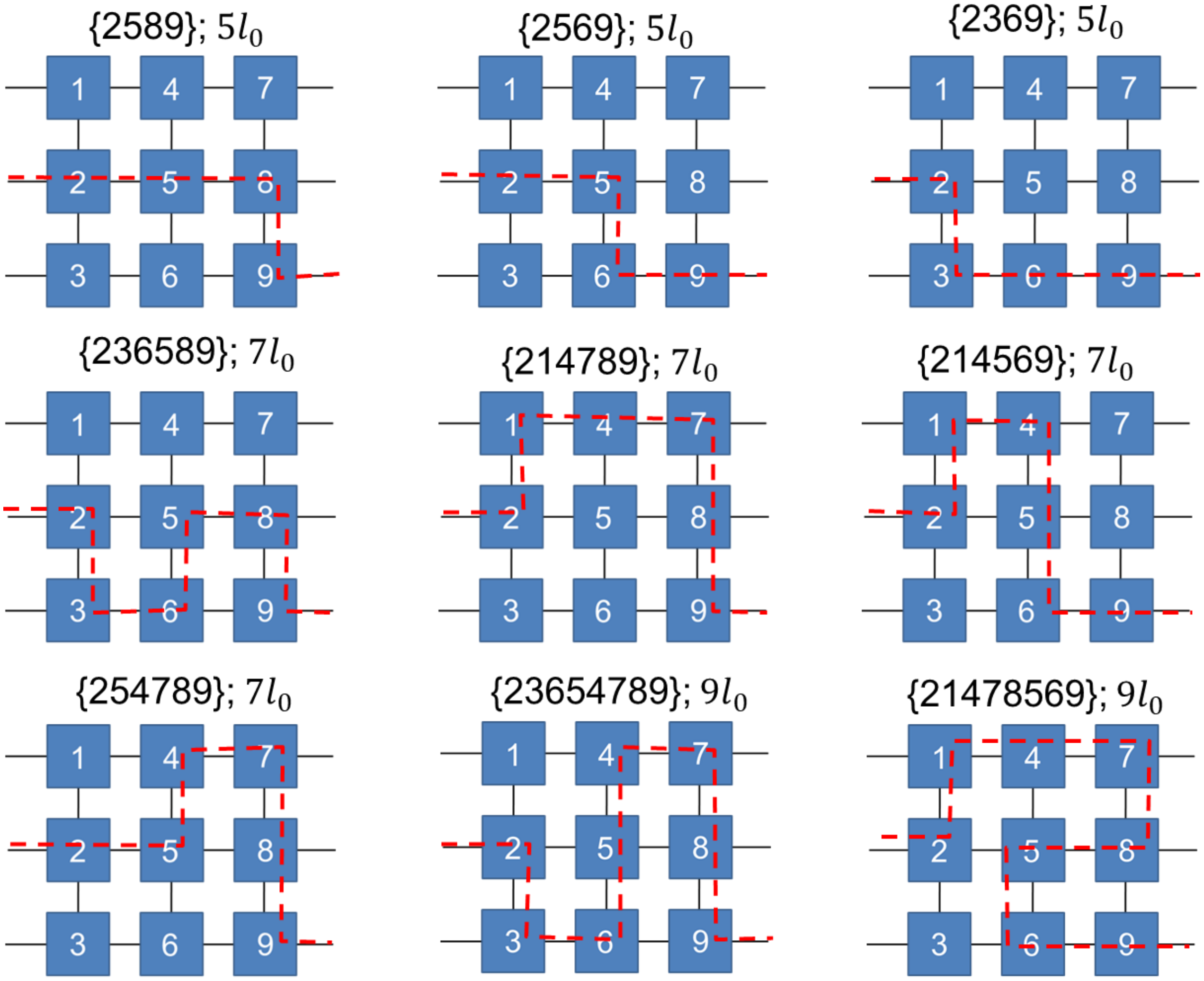

Schematics of all possible paths connecting input 3 and output 1

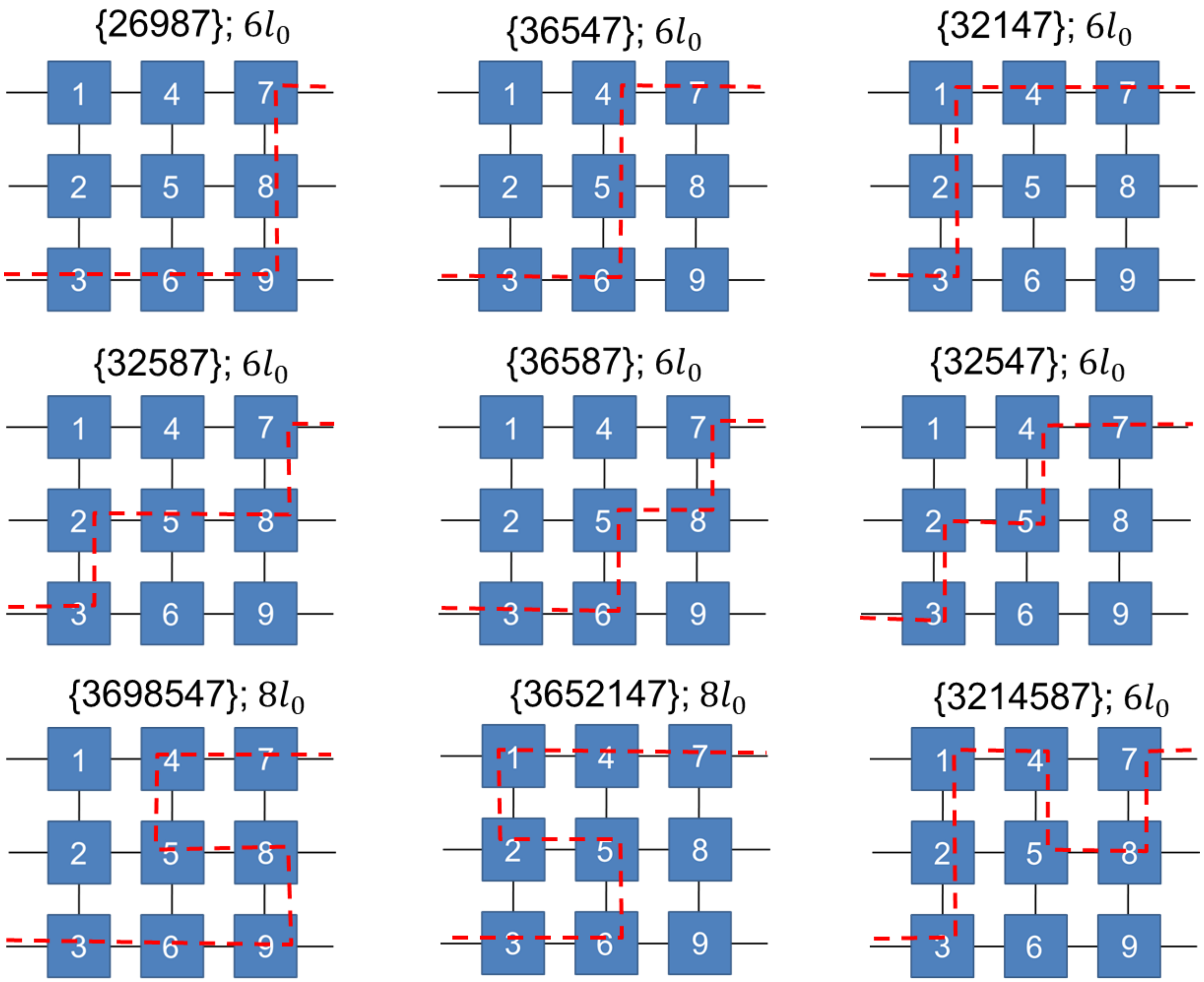

Schematics of all possible paths connecting input 3 and output 1 cont'd

{321456987}; $10l_0$

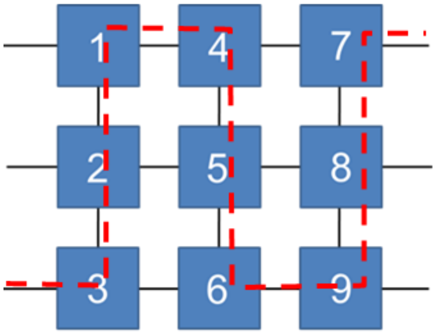

{369852147}; $10l_0$

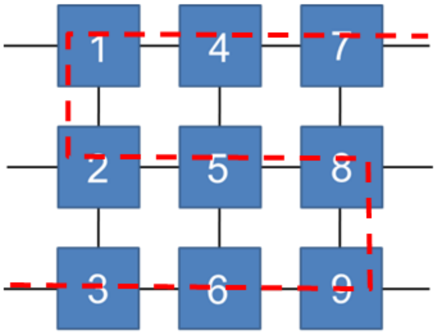

Schematics of all possible paths connecting input 3 and output 2

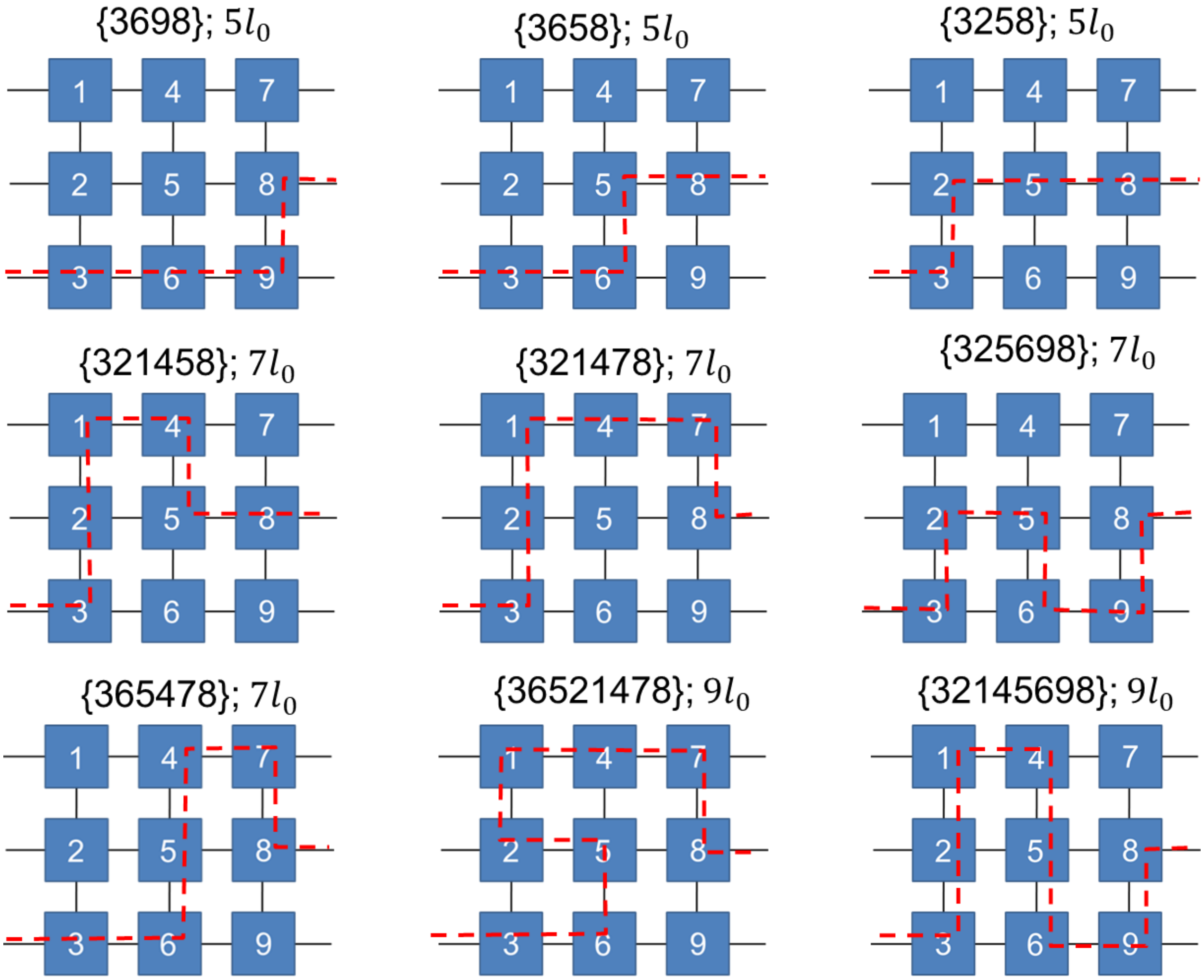

Schematics of all possible paths connecting input 3 and output 3

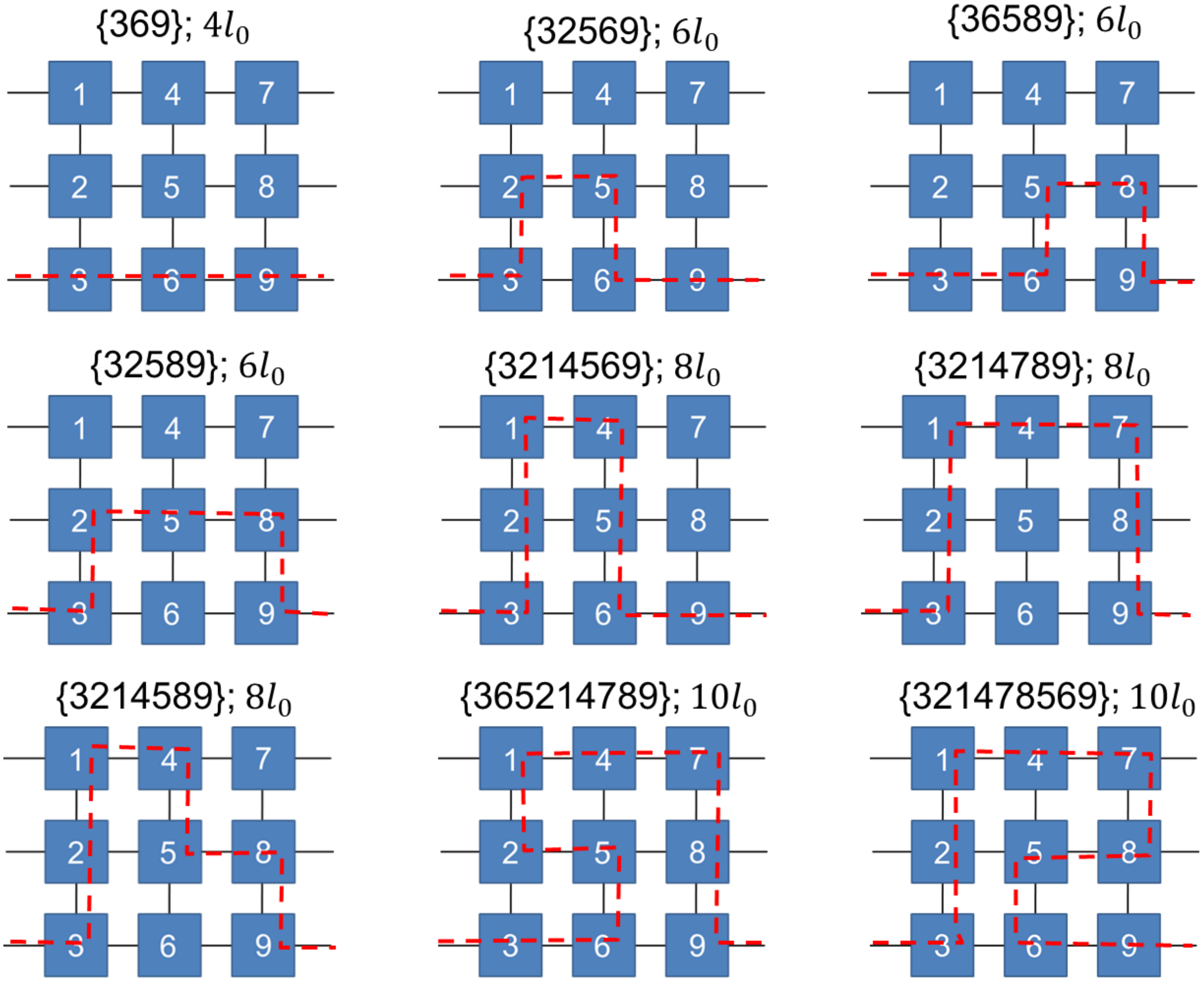

Supplement: Supplementary file 2 — Supplementary Information 2. [file 41598_2022_13614_MOESM2_ESM.pdf]
